# Vemurafenib in the Treatment of Erdheim Chester Disease: A Systematic Review

**DOI:** 10.7759/cureus.25935

**Published:** 2022-06-14

**Authors:** Syed N Aziz, Lucia Proano, Claudio Cruz, Maria Gabriela Tenemaza, Gustavo Monteros, Gashaw Hassen, Aakash Baskar, Jennifer M Argudo, Jonathan B Duenas, Stephanie P Fabara

**Affiliations:** 1 Internal Medicine, Shaheed Suhrawardy Medical College, Dhaka, BGD; 2 Internal Medicine, Pontificia Universidad Católica del Ecuador, Quito, ECU; 3 General Medicine, Universidad San Francisco de Quito, Quito, ECU; 4 School of Medicine, Universidad San Francisco de Quito, Quito, ECU; 5 General Medicine, Pontificia Universidad Católica del Ecuador, Quito, ECU; 6 Internal Medicine, University of Maryland Capital Region Medical Center, Largo, USA; 7 Medicine, Addis Ababa University, Addis Ababa, ETH; 8 Progressive Care, Mercy Medical Center, Baltimore, USA; 9 Medicine and Surgery, Parma University, Parma, ITA; 10 Medicine and Surgery, K.A.P Viswanatham Government Medical College, Tiruchirappalli, IND; 11 Child Neurology, Universidad de Cuenca, Cuenca, ECU; 12 Internal Medicine, Universidad Central del Ecuador, Quito, ECU; 13 Internal Medicine, North Florida Regional Medical Center, Gainesville, USA

**Keywords:** histiocytosis, neurology, macrophages, non-langerhans cell histiocytosis, erdheim chester disease

## Abstract

Erdheim Chester disease (ECD) is a type of histiocytosis characterized by a variable clinical presentation. The treatment of ECD is complex and mainly unknown. We aim to conduct a literature review of the treatment of ECD and consolidate the knowledge about the most recent and updated treatment for ECD. To conduct the systematic review, we used the preferred reporting items for systematic reviews and meta-analysis (PRISMA) protocol.

To analyze the bias, we used the Cochrane collaboration risk-of-bias tool to assess the bias. We included observational studies and clinical trials on humans, which were written in English. Papers not fulfilling the objective of our study were excluded.

Overall, the drug showed efficacy in the clinical trials, showing prolonged improvement and high rates of response rate. Overall, the drug was not well tolerated, and patients had a long list of side effects. Nevertheless, the drug seems to be a good option for second-line treatment for patients with ECD and BRAFV600 mutation.

## Introduction and background

A type of non-Langerhans cell histiocytosis was first described as lipoid granulomatosis in 1930 by Erdheim and Chester (Erdheim Chester disease; ECD) [1]. As a very rare disease, only about 1500 cases are documented until 2019 [2]. It is characterized by the accumulation and abnormal behavior of monocytes, histocytes, and macrophages [3]. The pathophysiology is mostly unclear. Studies found an imbalance between cytokines, mostly interleukin 1, interleukin 6, interferon-gamma, and interferon-alpha in patients with ECD [4]. Studies showed BRAF600E mutation in patients with ECD and also in patients with Langerhans cell histiocytosis [3,4].

Bone pain, usually in the leg, due to long bone osteosclerosis is the most common presentation of ECD [1]. The most commonly affected bones are the tibia and femur, and about 90% of ECD patients show bony involvement at some point in the disease [5]. Cardiovascular manifestation includes sheathing of the aorta, pericarditis, pericardial effusion, and cardiac tamponade. Right atrial pseudotumor is a classic for ECD [1,5]. Retroperitoneal fibrosis causing complicated bilateral hydronephrosis was found in one-third of patients [2]. Abdominal imaging revealed fat infiltration around the kidneys in more than half of the patients [1]. Pulmonary findings include subpleural or parenchymal nodule, an interlobar septal thickening mimicking interstitial lung disease [2,5]. Among endocrine manifestations of the disease, diabetes insipidus often appears as the earliest sign. Other hormonal abnormalities include growth hormone deficiency, thyrotropic deficiency, hyperprolactinemia, and gonadotropic deficiency [1,5]. Though adrenal infiltration was recorded in studies, adrenal deficiency was rare [2]. A xanthelasma-like lesion in the periorbital area is reported to be a common cutaneous feature [1]. Neurological manifestation of ECD is largely due to cerebellar and pyramidal syndromes. Besides, headache, seizure, cranial nerve palsy, neurocognitive impairment, and asymptomatic lesions are also recorded [1]. Retro-orbital infiltration causing exophthalmos has also been reported [5].

Biopsy from a skin lesion (e.g., xanthelasma) or from per-nephric fat infiltration (CT guided) can confirm the diagnosis of ECD [1]. Specimens are likely to be positive for one or more of these cell markers: CD68, CD163, or FXIIIa. While unless simultaneous involvement of Langerhans cell histiocytosis (LCH) is present, they should be negative for CD1a [5]. Long bone osteosclerosis of lower limbs is a defining characteristic of ECD, and thus PET CT of the whole body is a preferred study to find such lesions. Despite this, about 5% of patients may not have osteosclerotic lesions [6]. In the absence of typical features, molecular study like BRAFV600e mutation or mitogen-activated protein kinase (MAPK) pathway modification has become the gold standard for the diagnosis of ECD [1,6].

The treatment of ECD is mainly unknown; we aim to conduct a systematic review of the treatment of ECD and consolidate the knowledge about the most recent and updated treatment for ECD. The goal is to review the efficacy of the drug in ECD, and its safety.

## Review

Materials and methods

Protocol

The systematic review was performed via the preferred reporting items for systematic reviews (PRISMA) and meta-analysis [7].

**Figure 1:**
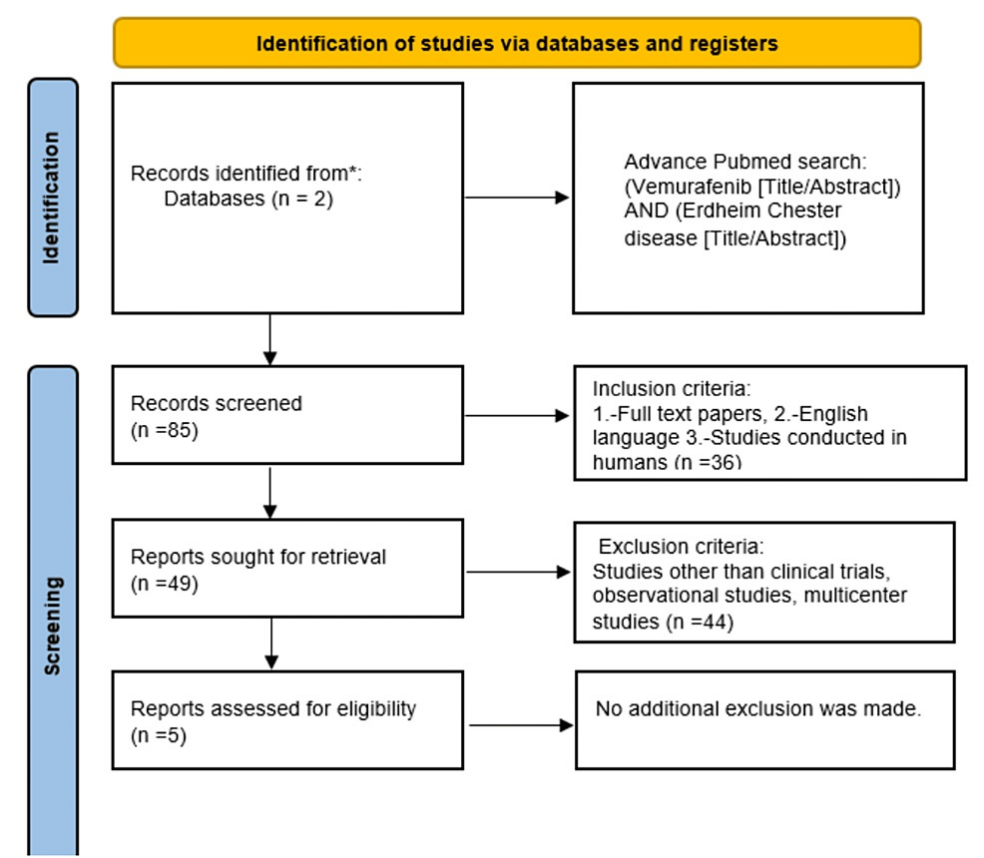
PRISMA flow chart of this systematic review We found five clinical trials discussing the role of vemurafenib in the treatment of ECD. ECD, Erdheim Chester disease.

Figure 1 shows the PRISMA flow chart of the systematic review.

Eligibility criteria and study selection: We included full-text observational studies and clinical trials on humans, which were written in the English language. Papers not fulfilling the objective of our study were excluded. After the screening process, we included papers with the following features:

(1) Patients: with ECD

(2) Intervention: use of vemurafenib

(3) Comparator: control placebo or control group

(4) Outcome: response rate and side effects

Database and search strategy: A systematic review was undertaken using a PubMed Database search between 1 April 2022 and 15 April 2022. We searched through advanced strategy using the following terms: (Vemurafenib [Title/Abstract]) AND (Erdheim Chester disease [Title/Abstract]).

Data extraction and analysis: We collected the following information from each paper: the author’s year of publication, number of patients in the treatment group, number of patients in the control group, selection of the patients, the efficacy of the drug, safety, and main conclusion.

Bias assessment: We used the Cochrane collaboration risk-of-bias tool to assess the bias encountered in each study [8].

Results

Study Characteristics

 Table 1 shows the methods of the studies for the systematic review [9-11].

**Table 1:** Main characteristics of each study. CNS, central nervous system; PET-CT, positron emission tomography and computed tomography; mg, milligrams.

Author and year of publication	Country	Study design	Number of patients in the treatment group	Number of patients in the control group	Patient selection	Dose, duration, and route of administration
Diamond et al. (2018) [9]	United States	Open-label, non-randomized, phase 2 clinical trials	22	No control group	Non-melanoma cancer patients with the BRAFV600 mutation	960 mg, every 12 hours continuously until perpetuation of disease, study withdrawal, or incident of unbearable adverse effects
Hyman et al. (2015) [10]	United States	Cohort	122	No control group	Patients holding a BRAFV600 mutation-positive non-melanoma cancer	960 mg, twice daily, oral dose
Haroche et al. (2015) [11]	United States	Open study	8	No control group	Patients with multisystemic Erdheim-Chester disease with CNS and/or cardiac involvement with BRAFV600 mutation	Patients 1, 2, and 3 were treated with vemurafenib 960 mg twice a day. Following this, treatment dosage was reduced after 30, 30, and 20 days in patients 1, 2, and 3, to 480 mg twice a day because of cutaneous adverse effects. Patient 4 had an initial dose of 960 mg twice a day; dosage was reduced after 5 days because of severe adverse effects. For the last four patients, treatment was initiated at 480 mg twice a day.

Study Outcome

Table 2 shows the outcomes and main conclusions of the studies [9-11].

**Table 2:** Study outcomes of the systematic review. ORR, objective response rate; PFS, progression-free survival; PET, positron-emission tomography; OS, overall survival; CT, computed tomography; response rate: 45% is high, 35% is low but desirable and indicates efficacy; AE, adverse effects; SUV, standardized uptake value; BCC, basal cell carcinoma; SCC, squamous cell carcinoma; FDG-PET/CT: fluorodeoxyglucose-positron emission tomography; ECD-LCH, Erdheim-Chester disease - Langerhans cell histiocytosis.

Author, year	Outcomes	Results of treatment group	Adverse effects	Results of control or placebo group	Main conclusion
Diamond et al. (2018) [9]	ORR by response evaluation criteria in solid tumors, PFS, overall survival, metabolic response by modified PET scan. Response criteria in solid tumors by using FDG-PET/CT safety	Patients with ECD had an ORR of 54.5% (95% CI, 32.2-75.6). PFS was 86% (95% CI, 72%-100%), and 2-year OS was 96% (95% CI, 87%-100%) (overall cohort). 80% of patients achieved a complete metabolic response, 20% got partial metabolic response; assessed via PET scan.	The most common AEs were rash, arthralgia, alopecia, fatigue, skin papilloma, hyperkeratosis, and prolonged QT interval. Eight patients discontinued vemurafenib due to AE.	There is not a placebo or control group	Vemurafenib had prolonged efficacy in patients with BRAFV600-mutant ECD and LCH. The drug has prolonged antitumor efficacy. The drug warrants consideration as a new standard of care for patients.
Hyman et al. (2015) [10]	Response rate at eight weeks. Progression-free survival. Overall survival. Safety	In the ECD-LCH cohort response rate was 43% (95% CI, 18-71). No patients with progression while on treatment. Preliminary 12-month PFS and OS were 91% and 100%, respectively. Safety was similar to previous studies on vemurafenib.	Common adverse effects were arthralgia, fatigue, and rash.	No control group	Vemurafenib had preliminary effect on BRAF600 (+) ECD-LCH. Further studies are needed to analyze the promising effect on the oncogene.
Haroche et al. (2015) [11]	Primary evaluation via PET response at six months. Secondary evaluation via comparing cerebral and cardiac MRI. Adverse effect.	All patients showed a partial metabolic response at 6 months of vemurafenib therapy, with a median reduction in SUVmax of 63.5%.	Seven out of eight patients with cardiac and aortic involvement showed partial response. MRI showed an objective decrease in infratentorial lesions in four patients. Keratosis pilaris, xerosis, photosensitivity, arthralgia, QT prolongation. One patient developed BCC and one developed SCC.	No control group	Despite severe cutaneous adverse effects, vemurafenib can be considered as a second-line therapy for BRAF600-mutated, INFα/anakinra-resistant ECD. Newer BRAF inhibitors should also be investigated for better tolerability.

Study Limitations

In the study of Diamond et al., there was no control group. So, there was no comparison between a treatment and non-treatment group. In the study, it was not stabilized about the optimal dose or duration of the treatment (vemurafenib) [9].

In Hyman et al.'s study, the small number of patients made it difficult to interpret the results of the study [10].

In the study of Haroche et al., only patients with failure to first-line treatment due to severe cutaneous adverse effects were chosen to be part of the study [11].

**Figure 2:**
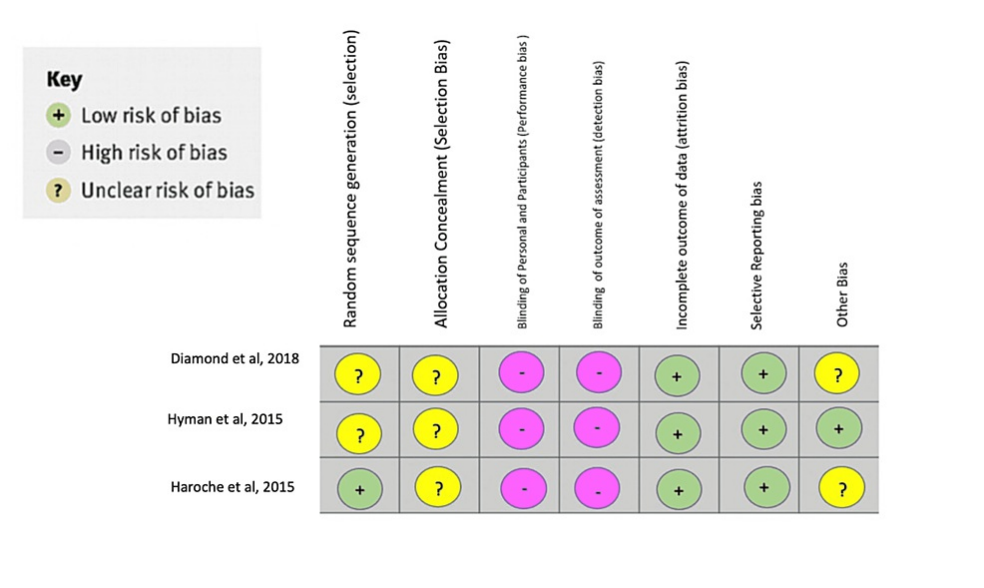
Bias analysis of the systematic review. Source: [9-11].

Figure 2 shows the bias of the systematic review [9-11].

Discussion

Vemurafenib is a potent oral highly selective inhibitor of mutated BRAFV600E (v-RAF murine sarcoma viral oncogene homolog B) [12]. Inhibiting the kinase activity of BRAF prevents signaling from the MAPK pathway and blocks the proliferation of malignant cells that harbor this specific mutation [13]. In 2017, the Food and Drug Administration (FDA) approved vemurafenib for the treatment of ECD, and the drug is also approved for metastatic and unresectable melanoma with V600 mutation, and ECD (non-Langerhans histiocytic disorder) [6,13]. The most common adverse events noticed with the use of vemurafenib are cutaneous manifestations and the most significant are skin cancers including squamous cell carcinoma and keratoacanthoma [14].

Clinical Trials

Hyman et al. showed that vemurafenib was efficacious, showing antineoplastic activity. The drug improved the response rate and halted the progression of the disease in patients with EDS that expressed BRAFV600 mutation in the phase clinical trial [10]. The results were statistically significant [10]. Safety was similar to previous studies. The most common AEs were rash, fatigue, and arthralgias [10]. Studies found that vemurafenib works on BRAF600-mutated non-small-cell lung cancer (NSCLC), but not on BRAFnon600 mutated NSCLC [15].

According to Haroche et al., ECD patients with BRAFV600 mutation who failed first-line treatment with interferon α were treated with vemurafenib, and all of them were partial metabolic responders [11]. The median reduction in SUV(max) was 63.5% for all patients, which was statistically significant [11]. Due to severe dermatologic adverse effects (SCC), one patient needed to stop vemurafenib [11]. Even though vemurafenib is an effective BRAFV600 inhibitor, newer drugs must be studied for better tolerability [11].

In the study of Diamond et al., 26 patients with BRAF mutations had a prolonged efficacy with a 62% response rate [9]. The results were statically significant. Overall, the drug was not well tolerated, with reported side effects such as skin papilloma, QT prolongation, maculopapular rash, fatigue, and hyperkeratosis. Higher rates of side effects were more common than in patients treated with metastatic melanomas [9]. In the same study, the drug proved effective for patients with LCH.

Overall, the studies do not have a control group, and the sample of studies was small, so the statistical power is small.

Neurological Manifestations' Response

ECD affects the central nervous system, causing the pyramid-cerebellar syndrome [15]. The main structures affected are the pons and red nucleus [15]. In a case report, a 31-year-old patient presented with diabetes insipidus, ataxia, hyperreflexia, skin xanthomas, and nystagmus. The patient was BRAFV600E positive, so vemurafenib was initiated [15]. During the first 10 months of treatment, there was a partial recovery, and after three years of treatment, the disease was halted [15].

Neurological manifestations are only seen in 37% of patients with ECD. The neurological manifestations are more frequent in BRAFV600E-mutated patients. Having neurological manifestation is also a sign of a bad prognosis [16]. Another patient had multiple manifestations, including a neurological picture of ataxia and lesions in the cerebellar peri-dentate region, the middle cerebellar peduncles, and the white matter of the pons. The patient was BRAF600 positive as well and had a dramatic response to the drug [17].

In another case, a man with a history of two months of headaches was present in the emergency department [18]. The man had decreased range of motion in the cervical spine and infiltrative lesions in the cerebellum, and parietal lobe, causing a mass effect, making the patient herniate. Neurosurgery operated on the patient, removing the mass and resolving the acute situation. The biopsy was positive for D68, CD163, Factor XIIIa, and BRAF, so the patient was put on vemurafenib, and continued to show improvement after a three-month follow-up [18].

More cases need to be analyzed, but the case report had a good response to the drug [16].

Orbital and Corioretinal Response

Orbital and chorioretinal manifestations are rare complications of the disease. In a case report, a patient with a delayed diagnosis developed this complication and was positive for BRAFV600E, so vemurafenib was initiated [19]. Orbital involvement can occur in 25-37%, and it presents with retrobulbar masses that generate impairment in the visual field, proptosis, optic edema, and motility disturbances [19]. In this case, chorioretinal manifestation did not improve, but visual acuity did improve, showing a limited benefit of the drug. The patient was also on vascular endothelial growth factor (VEGF) injection, so that drug could also be attributed to the benefit of the patient [19].

In two additional case reports, the first patient presented with a mix of orbital and neurological manifestations, and the patient had an indolent course of the disease. The treatment was initiated after two years of diagnosis, resulting in the reversal of vision loss and recovery from the other systemic manifestations [20]. In the second case, the disease course was more aggressive, the patient lost sight in six weeks, and treatment with vemurafenib was unable to prevent blindness in this patient [20].

Orbital and chorioretinal manifestations have been shown to be a bad prognosis factor. The manifestations have shown variable responses to VEGF injections, so new treatments are necessary to halt the progression of the symptoms and improve the clinical picture in patients with orbital and chorioretinal manifestations [19].

## Conclusions

It can be concluded based on our review data that patients with ECD responded to vemurafenib treatment. Vemurafenib showed antineoplastic activity, improving the response rate and halting the disease progression in the clinical trials. An inconvenience with the trials was the lack of a control group. Future studies should benefit to have a placebo or control group.

Overall, the drug was not well tolerated, and patients had many side effects, with the most common being the cutaneous ones. Nevertheless, the drug seems to be a good option as a second-line treatment for ECD patients with BRAFV600 mutation.

Several case reports showed improvements in neurological, orbital, and chorioretinal manifestations for patients with BRAFB600 mutation, who had vemurafenib later in their treatment course. Nevertheless, the significance of those findings is small due to the study types (case reports).

Future studies with larger sample sizes and control groups are required to properly evaluate the promising role of vemurafenib in defining the efficacy and the safety of the drug.

## References

[1] Haroche J, Cohen-Aubart F, Amoura Z (2020). Erdheim-Chester disease. Blood.

[2] Papo M, Emile JF, Maciel TT (2019). Erdheim-Chester disease: a concise review. Curr Rheumatol Rep.

[3] Cangi MG, Biavasco R, Cavalli G (2015). BRAFV600E-mutation is invariably present and associated to oncogene-induced senescence in Erdheim-Chester disease. Ann Rheum Dis.

[4] Munoz J, Janku F, Cohen PR, Kurzrock R (2014). Erdheim-Chester disease: characteristics and management. Mayo Clin Proc.

[5] Starkebaum G, Hendrie P (2020). Erdheim-Chester disease. Best Pract Res Clin Rheumatol.

[6] Goyal G, Heaney ML, Collin M (2020). Erdheim-Chester disease: consensus recommendations for evaluation, diagnosis, and treatment in the molecular era. Blood.

[7] Moher D, Liberati A, Tetzlaff J, Altman DG (2009). Preferred reporting items for systematic reviews and meta-analyses: the PRISMA statement. PLoS Med.

[8] Higgins JP, Altman DG, Gøtzsche PC (2011). The Cochrane Collaboration's tool for assessing risk of bias in randomised trials. BMJ.

[9] Diamond EL, Subbiah V, Lockhart AC (2018). Vemurafenib for BRAF V600-mutant Erdheim-Chester disease and Langerhans cell histiocytosis: analysis of data from the histology-independent, phase 2, open-label VE-BASKET study. JAMA Oncol.

[10] Hyman DM, Puzanov I, Subbiah V (2015). Vemurafenib in multiple nonmelanoma cancers with BRAF V600 mutations. N Engl J Med.

[11] Haroche J, Cohen-Aubart F, Emile JF (2015). Reproducible and sustained efficacy of targeted therapy with vemurafenib in patients with BRAF(V600E)-mutated Erdheim-Chester disease. J Clin Oncol.

[12] Donadieu J, Larabi IA, Tardieu M (2019). Vemurafenib for refractory multisystem Langerhans cell histiocytosis in children: an international observational study. J Clin Oncol.

[13] Khaddour K, Kurn H, Zito PM (2022). Vemurafenib. https://www.ncbi.nlm.nih.gov/books/NBK535429/.

[14] Mazieres J, Cropet C, Montané L (2020). Vemurafenib in non-small-cell lung cancer patients with BRAFV600 and BRAFnonV600 mutations. Ann Oncol.

[15] Fernández-Eulate G, Muñoz-Lopetegi A, Ruiz I, Urtasun M (2019). Vemurafenib as first-line therapy in BRAF-V600E-mutant Erdheim-Chester disease with CNS involvement. BMJ Case Rep.

[16] Cohen-Aubart F, Emile JF, Carrat F (2018). Phenotypes and survival in Erdheim-Chester disease: results from a 165-patient cohort. Am J Hematol.

[17] Mazor RD, Manevich-Mazor M, Kesler A (2014). Clinical considerations and key issues in the management of patients with Erdheim-Chester disease: a seven case series. BMC Med.

[18] Fasulo S, Alkomos MF, Pjetergjoka R (2021). Erdheim-Chester disease presenting at the central nervous system. Autops Case Rep.

[19] Huang LC, Topping KL, Gratzinger D, Brown RA, Martin BA, Silva RA, Kossler AL (2018). Orbital and chorioretinal manifestations of Erdheim-Chester disease treated with vemurafenib. Am J Ophthalmol Case Rep.

[20] Brodie J, Zhou S, Makkuni D (2020). Erdheim-Chester disease: two cases from an ophthalmic perspective. Am J Ophthalmol Case Rep.

